# Thermoplastic Dynamic Vulcanizates with In Situ Synthesized Segmented Polyurethane Matrix

**DOI:** 10.3390/polym11101663

**Published:** 2019-10-12

**Authors:** Andrea Kohári, István Zoltán Halász, Tamás Bárány

**Affiliations:** Department of Polymer Engineering, Faculty of Mechanical Engineering, Budapest University of Technology and Economics, Műegyetem rkp. 3., H-1111 Budapest, Hungary; koharia@pt.bme.hu (A.K.); halaszi@pt.bme.hu (I.Z.H.)

**Keywords:** thermoplastic dynamic vulcanizates, TDV, thermoplastic polyurethane, TPU, in situ produced matrix

## Abstract

The aim of this paper was the detailed investigation of the properties of one-shot bulk polymerized thermoplastic polyurethanes (TPUs) produced with different processing temperatures and the properties of thermoplastic dynamic vulcanizates (TDVs) made by utilizing such in situ synthetized TPUs as their matrix polymer. We combined TPUs and conventional crosslinked rubbers in order to create TDVs by dynamic vulcanization in an internal mixer. The rubber phase was based on three different rubber types: acrylonitrile butadiene rubber (NBR), carboxylated acrylonitrile butadiene rubber (XNBR), and epoxidized natural rubber (ENR). Our goal was to investigate the effect of different processing conditions and material combinations on the properties of the resulting TDVs with the opportunity of improving the interfacial connection between the two phases by chemically bonding the crosslinked rubber phase to the TPU matrix. Therefore, the matrix TPU was synthesized in situ during compounding from diisocyanate, diol, and polyol in parallel with the dynamic vulcanization of the rubber mixture. The mechanical properties were examined by tensile and dynamical mechanical analysis (DMTA) tests. The morphology of the resulting TDVs was studied by atomic force microscopy (AFM) and scanning electron microscopy (SEM) and the thermal properties by differential scanning calorimetry (DSC). Based on these results, the initial temperature of 125 °C is the most suitable for the production of TDVs. Based on the atomic force micrographs, it can be assumed that phase separation occurred in the TPU matrix and we managed to evenly distribute the rubber phase in the TDVs. However, based on the SEM images, these dispersed rubber particles tended to agglomerate and form a quasi-continuous secondary phase where rubber particles were held together by secondary forces (dipole–dipole and hydrogen bonding) and can be broken up reversibly by heat and/or shear. In terms of mechanical properties, the TDVs we produced are on a par with commercially available TDVs with similar hardness.

## 1. Introduction

One of the most important reasons for the successful and continuously growing market penetration of thermoplastics is the diverse characteristics of their blends. By melt blending two (or more) different polymers, a material with new or improved properties can be produced. Blending can enhance mechanical performance (especially toughness), resistance to thermal degradation, improve processability, support cost efficiency, etc., or even result in novel properties. One emerging family of polymer blends is the group of thermoplastic dynamic vulcanizates (TDVs). These TDVs, composed of a continuous thermoplastic phase in which a dynamically cured rubber phase is dispersed, combine the elasticity of rubbers with the easy processing and recyclability of thermoplastics. The term “dynamically” refers to the fact that the rubber component is cured simultaneously with its dispersion in the molten thermoplastic resin by intensive mixing/kneading [1,2,3]. With this technique, a fine dispersion of the rubber phase in the thermoplastic matrix can be achieved, which is often referred to as a “sea-island” structure with submicron–micron-sized rubber “islands” in the thermoplastic matrix “sea”. This structure combines the beneficial properties of the components, namely, the elastic behavior of rubbers and the simple processing (and possible reprocessing as well) of thermoplastic polymers [4].

The development of TDVs started with research on the impact modification possibilities of isotactic polypropylenes (iPP). The results of these studies clarified that the impact resistance of iPP homopolymers could be highly improved through the incorporation of uncured ethylene-propylene-based rubbers. This toughening was even more pronounced below the glass transition temperature range of iPP and was strongly dependent on the dispersion state of the rubber phase. However, the dispersibility of the rubber phase was limited by the recurring agglomeration and coalescence of the rubber particles, which happened during compounding. As a solution for this issue, partial curing of the rubber phase was recommended and attempted [5,6,7]. The breakthrough was achieved in the late 70s and early 80s when several patents were filed and the first commercialized TDV appeared under the trademark of Santoprene^®^ by Monsanto. It was composed of iPP and ethylene-propylene-diene terpolymer rubber (EPDM). Based on intensive research, the requirements for the optimal mechanical performance of TDVs were deduced, which can be summarized as follows [8,9,10]:

- the interfacial tension of the constituents is small,
- the crosslink density of the rubber phase is relatively high,
- the matrix polymer is semi-crystalline [11,12].

Similar to blends of various polymers, polyurethanes (PUs) can satisfy an enormously wide range of application requirements due to the great structural diversity of its constituents: isocyanates, polyols, and chain extenders. These constituents may be bifunctional or polyfunctional, allowing the production of both linear and crosslinked molecular structures. Through the appropriate selection of the properties of the precursors such as the polarity of the chains, the mechanical, thermal, and optical behavior of the final PU can be tailored according to the requirements, and through the modification of the constituents, reactive moieties can be introduced into the final chain [13,14,15,16]. PUs can be synthetized in one shot or via the pre-polymer route. The former means the simultaneous mixing of all three main PU components, while the latter is composed of two separate steps. In the first step, the polyol reacts with an excess of isocyanate to form an isocyanate-terminated urethane prepolymer, then this prepolymer reacts with the chain extender and the PU is obtained [17]. The one-shot method leads to a relatively more random polymer chain segment structure, whereas the prepolymer route delivers a more regular sequence of the chain segments [18].

Several studies can be found on compounding PUs with rubbers (particularly with NBR rubbers, due to their similar polar character) with the aim of producing polymer blends with novel performance characteristics, although these usually focus on solution and high-temperature melt blending techniques, which are less economical as they need more time and energy. For this reason, dynamic curing is seldom, if ever, used. It was reported that the quasi-static and dynamic mechanical properties were improved (sometimes even outperforming the neat PU), although this reinforcing effect was limited by the melting of the PU phase at higher temperatures as well as by the breakdown of secondary structures between the PU and the NBR at higher strain amplitudes [19]. The same reinforcing effect was observed in blends produced with the solution method [20]. It was reported that if the acrylonitrile content of the NBR was increased, the properties of the blends improved due to the better compatibility between the PU and NBR phases [21]. The incorporation of PU in NBR and hydrogenated NBR (HNBR) rubbers were also researched with the aim of enhancing the wear resistance of the rubber. This technique may have application potential in areas where elastic behavior and good wear resistance are simultaneously required [11,12].

As introduced above, materials with promising novel performance characteristics can be created by blending thermoplastic polymers with rubbers. This potential can be augmented by the utilization of thermoplastic polyurethanes as matrix materials due to their relatively simple in situ synthesis. Note that a wide range of properties can be obtained within the thermoplastic PU family due to the diversity of the available precursors. Additional benefits can be expected from the dynamic curing introduced above. Our present paper represents the first steps of our research in this field and is aimed at investigating the properties of one-shot bulk polymerized TPUs produced with different processing temperatures as well as the properties of TDVs produced by the dynamic vulcanization of various rubber compounds accompanied by the in situ polymerization of the TPU matrix. The target of this research work was the exploration of the effect of different processing conditions and the material combination on the properties of the resulting TDV, keeping the mechanical performance in focus.

## 2. Materials and Methods

### 2.1. Materials and Processing

The tested polyurethanes were prepared from 4,4′-methylenebis(phenyl isocyanate) (MDI), 1,4-butanediol chain extender (BD), and polyether polyol poly(tetrahydrofuran) (PTHF) with a molecular mass of 1000 g/mol and a functionality of 2.0. The purity of all components was over 99%; the components were supplied by Sigma-AldrichDarmstadt, Germany. MDI was used as received, and butanediol and PTHF were dried at 90 °C for 4 h in vacuum. The composition of the materials was described by the ratio of the –NCO (isocyanate) and –OH (hydroxyl) functional groups at the start of the reaction (NCO/OH ratio) and the ratio of the –OH functional groups of the polyol to the total diol (pOH/OH ratio). The NCO/OH ratio was kept constant at 1.05, while the pOH/OH ratio of the TPUs was set to 0.5.

As a base rubber for the rubber phase of the TDVs acrylonitrile-butadiene (NBR), carboxylated acrylonitrile-butadiene (XNBR), and epoxidized natural rubber (ENR) were used. The NBR rubber was produced by Lanxess under the name of Perbunan 3445F. Its Mooney viscosity (ML, 1 + 4, 100 °C) was 45 ± 5, and its bound acrylonitrile content was 34 ± 1 wt%. The XNBR rubber was produced by Lanxess under the name of Krynac X146 with a Mooney viscosity (ML, 1 + 4, 100 °C) of 45 ± 5 and a bound acrylonitrile content of 32.5 ± 1.5 wt%. The ENR rubber was produced by Muang Mai Guthrie Company Limited under the name of Dynathai Epoxyprene 50. Its Mooney viscosity (ML, 1 + 4, 100 °C) is 70–90 and its level of epoxidation is 50 ± 2%. As a curative, dicumyl peroxide with 40 wt% active peroxide content (DCP40) was used (Norac, Norox DCP-40BK). The formulation of the rubber mixtures was as follows: base rubber 100 phr, DCP40 3.75 phr (1.5 phr active peroxide). The rubber mixtures were prepared on a laboratory two-roll mill (Labtech LRM-SC-110, Labtech Engineering Co. Ltd., Samutprakarn, Thailand) at roll temperatures of 70 and 50 °C (front and rear, respectively), and friction of 1.3.

**Figure 1:**
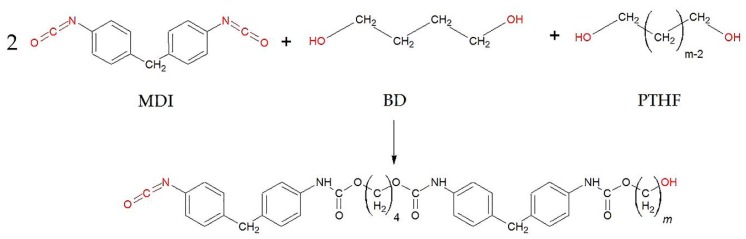
The reaction scheme of the synthesis of thermoplastic polyurethane.

One-step bulk polymerization of the TPUs and dynamic vulcanization of the rubber phase was carried out in a Brabender internal mixer (Brabender Plasti-Corder equipped with a W 50 EHT chamber, Brabender GmbH., Duisburg, Germany) at initial temperatures of 100, 110, 125, and 150 °C for the TPU production and 125 °C for the TDV production with a rotor speed of 50 rpm and a mixing time of 30 min. The reaction scheme is shown in Figure 1. Torque and melt temperature were recorded during the process. TDVs contained 50 wt% TPU and 50 wt% rubber mixture. The initial temperature was 125 °C. First, the components of the TPU were added, then the rubber blend after 13 min.

The 0.5 mm thick TPU and TDV sheets were compression molded at 190 °C under a pressure of 2 MPa for 3 min in a Collin Teach-Line Platen Press 200E laboratory press (Dr. Collin GmbH, Ebersberg, Germany). Specimens for further tests were cut from the sheets produced.

### 2.2. Testing Methods

Hardness was determined according to the ISO 7619-1 Shore A method with a Zwick H04.3150.000 hardness tester (Zwick GmbH, Ulm, Germany) on the compression molded sheets. Ten tests were performed on each compound.

The tensile mechanical properties of the compounds were investigated according to the ISO 37 standard on a Zwick Z005 universal testing machine with a 5 kN load cell (Zwick GmbH, Ulm, Germany) at room temperature at a crosshead speed of 500 mm/min. Five tests were performed on each compound.

Curing properties of the rubber compounds were tested on a MonTech D-RPA 3000 Dynamic Rubber Process Analyzer, (MonTech GmbH, Buchen, Germany). The curing curves were recorded both in isothermal conditions at 170 °C (1.67 Hz and 1° amplitude) and non-isothermal conditions. The purpose of the latter was to verify the proper vulcanization of the rubber phase during dynamic vulcanization, therefore the temperature profile of the vulcanization test was set according to the temperature curves recorded during the production of the corresponding TDV.

Polymerization of the TPU phase was investigated with a TA AR 2000 parallel plate rheometer (TA Instruments Ltd., New Castle, DE, USA) with a sinusoidal oscillation. We set the temperature profiles of each test according to the temperature curves recorded during the production of the TDVs in order to confirm the proper polymerization of the TPU. The strain amplitude and oscillation frequency were 1% and 10 rad/s. Curves were recorded on a time scale of 30 min and the gap was set to 0.5 mm between the parallel plates, which had a diameter of 25 mm.

Thermal analysis of the samples was carried out on a TA Q2000 DSC machine (TA Instruments Ltd., New Castle, DE, USA) in a N_2_ atmosphere with a heat–cool–heat cycle. The heating rate was 10 °C/min in the −90….250 °C range.

Dynamic mechanical tests of the compounds were performed with a TA Q800 DMTA machine (TA Instruments Ltd., New Castle, DE, USA) in tensile mode on rectangular specimens with dimensions of 0.5 mm × 2.5 mm × 10 mm (thickness × width × clamped length). Tests were run in the range of −100…200 °C with a 3 °C/min heating rate at a frequency of 10 Hz with a preload of 0.01 N and superimposed 0.1% sinusoidal strain.

The structure of the TPUs and TDVs were analyzed by attenuated total reflectance Fourier transform infrared spectroscopy (ATR-FTIR). A Bruker Tensor type FTIR machine (Bruker Optics Inc., Billerica, MA, USA) was used equipped with a Specac Golden Gate ATR unit (Specac Ltd., Orpington, UK) in the wavelength range of 4000 to 600 cm^−1^ with a resolution of 4 cm^−1^, accumulating 16 scans.

The morphology of the TDVs was characterized with the use of AFM micrographs of cryo-microtomed surfaces. Samples were cooled down to −80 °C and cut with a glass knife with the use of a Leica EM UC6/FC7 ultramicrotome (Leica Microsystems GmbH, Wien, Austria). AFM images were taken with a Nanosurf FlexAFM 5 (Nanosurf AG, Liestal, Switzerland) type AFM in tapping mode in air at room temperature. A single-beam silicon cantilever was used with a nominal force constant of 48 N/m and a resonance frequency of 190 kHz. It was a TAP 190Al-G cantilever (Budget Sensors, Innovative Solutions Bulgaria Ltd., Sofia, Bulgaria). The scan rate was between 0.5 and 1.0 Hz. The morphology of the TDVs was also investigated with a JEOL JSM 6380LA (Jeol Ltd., Tokyo, Japan) type scanning electron microscope (SEM). The samples were immersed in liquid nitrogen for 3 min and then fractured. The fractured surfaces were etched with boiling tetrahydrofuran (good solvent for TPU) for 1 h. After that, the samples were dried under vacuum for a day [22]. Before the test, the etched surfaces were coated with a thin layer of gold.

## 3. Results

### 3.1. Synthesis of Thermoplastic Polyurethanes (TPUs)

**Figure 2:**
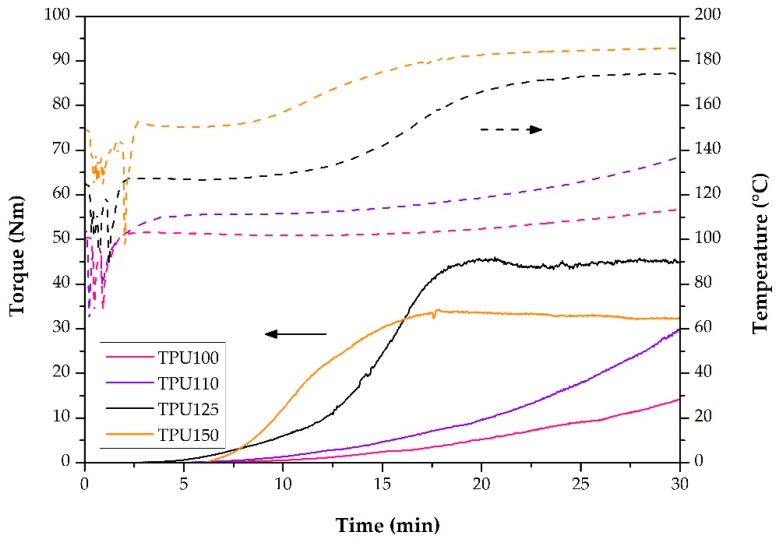
The parameters recorded during the synthesis of the thermoplastic polyurethanes (TPUs): torque curves (–––) and temperature curves (– – –).

First, we evaluated the torque and temperature curves that were recorded during the production of the specimens. The temperature profiles are very important because we needed to select an initial temperature in which the TPU could be polymerized and the rubber phase could be vulcanized at a sufficient rate, but without the degradation of the materials. The torque curves measured during the reactions also carry important information. Torque is proportional to melt viscosity, which depends on molecular weight. Thus, the torque change in the internal mixer is a good indicator of the change in molecular weight [23]. Figure 2 shows that in the case of the samples with initial temperatures of 100 and 110 °C, the torque increased until the end of the mixing time and did not reach an equilibrium. From this, we can conclude that polymerization was too slow at these initial temperatures, and did not finish within the time span of compounding. In contrast, at the initial temperatures of 125 and 150 °C, the torque reached a plateau. Due to the nature of the step polymerization in the first two cases (100 and 110 °C), oligomers with various molecular weights or perhaps short polymers chains were produced, while in the second two cases (125 and 150 °C), high molecular weight polymer chains were generated at the end of the mixing time. Figure 2 also shows the recorded temperature curves. In the case of the TPU100 and TPU110 samples, the temperature curves also did not reach a steady state. The maximum temperatures (114 and 137 °C) were low for the vulcanization of the rubber phase, and polymerization was also slow at these temperatures. In contrast, the 186 °C of the TPU150 sample could lead to the degradation of the rubber phase. The final temperature of the TPU125 sample was 175 °C, which is suitable for both polymerization and vulcanization, so we chose this initial temperature (125 °C) for preparing the TDVs.

### 3.2. Thermoplastic Dynamic Vulcanizates (TDVs)

#### 3.2.1. Production of TDVs

##### Isothermal Curing Properties

**Figure 3:**
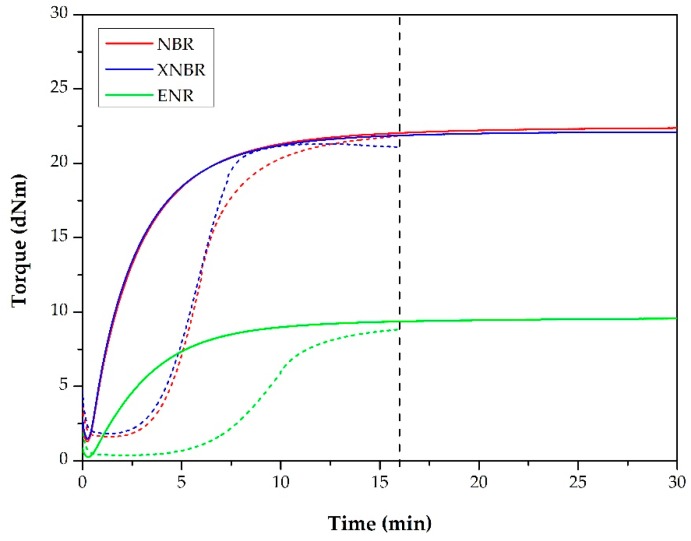
The curing curves of the rubber mixtures in isothermal (–––) and in non-isothermal conditions (– – –).

Before preparing the TDVs, we determined the isothermal curing properties of the rubber mixtures (continuous lines in Figure 3). It can be observed that in the case of NBR and XNBR, the curves almost ran together, but in the case of ENR, the torque was far lower during the tests. We determined the vulcanization times (*t*_90_) of the mixtures, which was 7 min for NBR and XNBR, and 8 min for ENR.

On the basis of the torque curves of the TPUs (Figure 2) and the vulcanization times, we determined that we had to add the rubber mixtures in the thirteenth minute of mixing to have in situ polymerization and vulcanization occur simultaneously.

##### Torque and Temperature Curves

**Figure 4:**
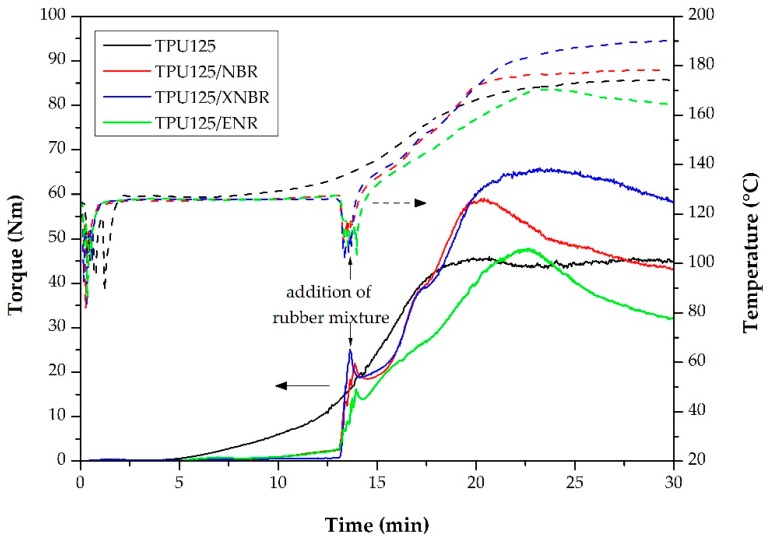
The parameters recorded during the synthesis of the thermoplastic dynamic vulcanizates (TDVs): torque curves (–––) and temperature curves (– – –).

The best way to keep track of the processes in the internal mixer is to analyze the torque and the temperature curves. In the torque curves recorded during the production of TDVs (Figure 4), there was a sharp and steep increase when the rubber mixture was added, followed by a sudden decrease. This decrease was due to the mastication of the rubber. Thereafter, the curves showed a pronounced increase again, which was due to the polymerization of the TPU and the vulcanization of the rubber phase. Finally, in the last stage, there was another decrease due to the fragmentation and dispersion of the vulcanized rubber islands.

The temperature curves (Figure 4) decreased at two points where we added the components. Figure 4 clearly shows that the torque and temperature values of the TDV sample containing ENR were well below that of the other two samples as well as the matrix TPU. The ENR rubber mixture was much softer than the others, consequently, even after the addition of ENR, the torque did not increase as intensely as in the internal mixer. At the same time, the temperature increase due to friction was also reduced, therefore we did not achieve the optimal conditions for the vulcanization of the rubber phase.

##### Non-Isothermal Curing Properties

**Figure 5:**
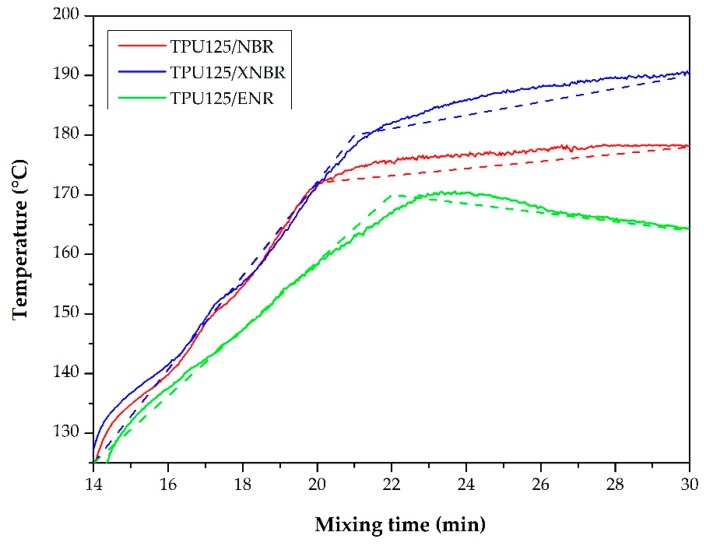
The real temperature profiles recorded during the production of TDVs (–––) and their approximate curves (– – –) that were used for the non-isothermal curing test for the rubber compounds.

Later, we also recorded the non-isothermal curing curves of the mixtures (Figure 3, dashed lines). The purpose of the test was to verify whether the rubber phase was properly vulcanized, therefore the temperature profile of the test was set according to the temperature curves recorded during the production of the corresponding TDV. We approximated these temperature curves with linear sections as shown in Figure 5.

About 16 min were available for the vulcanization of the rubber mixtures during the production of TDVs, which we indicated with a vertical line in Figure 3. The non-isothermal curing test indicated that we achieved quite a good degree of vulcanization in TDVs containing NBR and XNBR. As shown in Figure 3 (dashed line), the torque curve of the ENR sample did not show saturation. This is because the maximum temperature that the TDV containing ENR reached was not sufficient to completely vulcanize the rubber phase during compounding. This is also supported by the fact that the material removed from the internal mixer was in this case slightly tacky.

##### The Investigation of Matrix Polymerization

**Figure 6:**
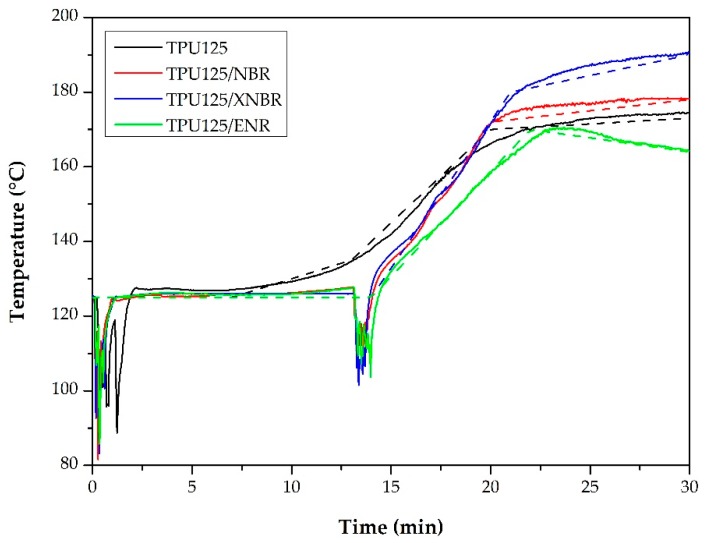
The real temperature profiles recorded during the production of TDVs (–––) and the approximate curves (– – –) that were used for the investigation of the polymerization tests of the matrix TPU.

In a rheometer, we modeled the processes in the internal mixer in order to investigate the polymerization of the matrix. Our aim was verify that the polymerization of TPU is possible with the temperature profiles of the production of TDVs. We approached these temperature curves with straight sections as shown in Figure 6 (dashed lines).

**Figure 7:**
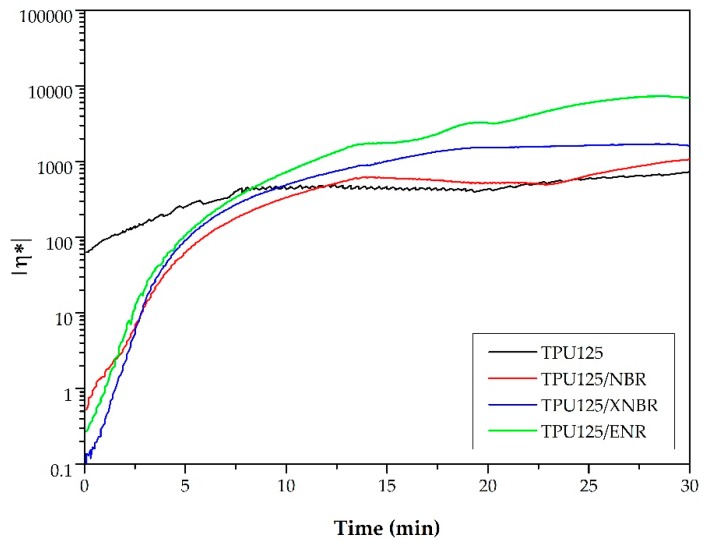
The simulation of the complex viscosity of the matrices in a rheometer during the production of pure TPU and TDVs.

During the evaluation of the curves, we examined the complex viscosity (η*) of the TPU. Based on this, we compared the processes during the synthesis of pure TPU and those during the production of TDVs. The curves (Figure 7) show that the thermoplastic polyurethane polymerized in each TDV because the final complex viscosity values are similar or greater than those of the TPU.

#### 3.2.2. Mechanical Properties

**Figure 8:**
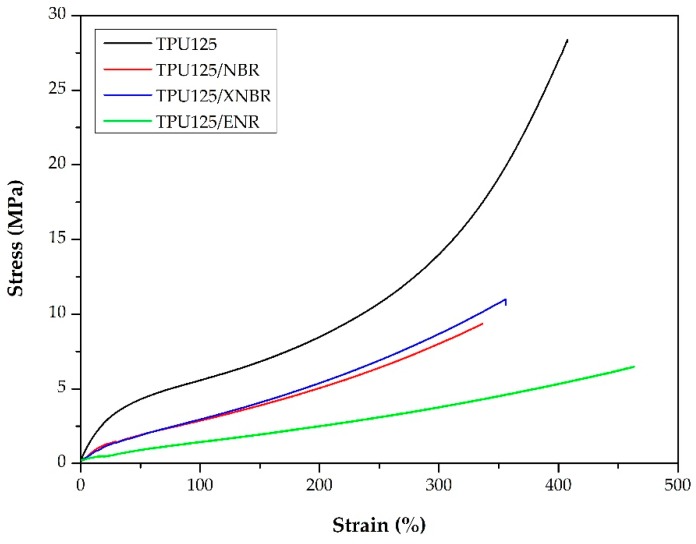
Typical tensile curves of the TPU matrix and the TDVs.

The tensile test results show that the modulus (Figure 8), the tensile strength, and the elongation at break of the TDVs are lower than those of the pure TPU (Table 1). The hardness of the materials was reduced by the rubber phase, which is consistent with the decrease in modulus. TDVs containing NBR and XNBR have similar strength properties and Shore A hardness. The tensile strength and hardness of the TPU125/ENR sample are lower, while the elongation at break is higher than for other TDVs.

These results are due to the poor properties of the cured rubbers. Our rubber blends were only model materials that did not contain any filler (e.g., carbon black, silica) and thus had poor mechanical properties. We used such model mixtures because at this stage of our research, we sought simplicity to minimize the effects of possible variables and influencing parameters.

#### 3.2.3. Dynamical Mechanical Analysis

**Figure 9:**
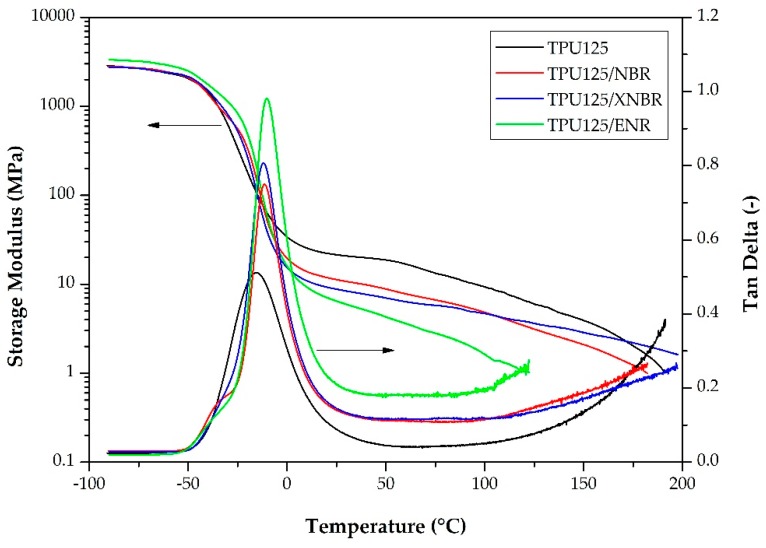
Thermomechanical curves of the TPU matrix and the TDVs.

Figure 9 and Table 2 show that the *T*_g_ values of the TDVs are higher than those of the matrix. On the tan delta curves of the TDVs, there is a shoulder on the damping peak, which belongs to the glass transition temperature of the soft segments of the TPU phase, while the well-developed peak represents the glass transition of the rubber phase.

In accordance with the results of the tensile test, the storage moduli of the TDVs are smaller than that of the TPU matrix, which is caused by the inherently lower storage modulus of the rubbers when compared to the TPU matrix.

#### 3.2.4. Differential Scanning Calorimetry

**Figure 10:**
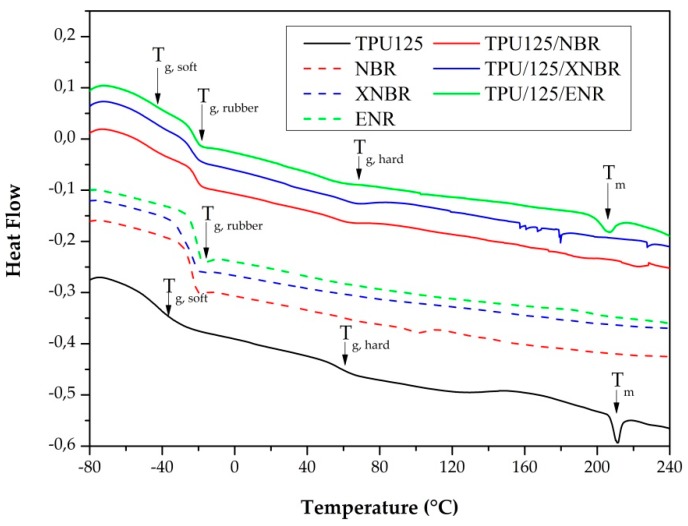
DSC thermograms of the TPU matrix and the TDVs.

On the thermograms of the TDVs, there are three different glass transitions. The first one belongs to the soft segments of the TPU, the second to the rubber phase, and the third to the TPU′s hard segment (Figure 10 and Table 3). On the curve of TPU125 and TPU125/ENR, there is also a melting peak, which belongs to the crystalline part of the hard phase. The melting temperature and the peak intensity of the TPU125/ENR sample are smaller than those of the TPU125. For the other two samples, the melting peak is not visible. This is due to the rubber phase, which did not allow the TPU chains to organize and crystallize because the hard segments can form strong hydrogen bonds with the –C≡N and >C=O groups of the rubber [24].

#### 3.2.5. Fourier-Transform Infrared Spectroscopy

**Figure 11:**
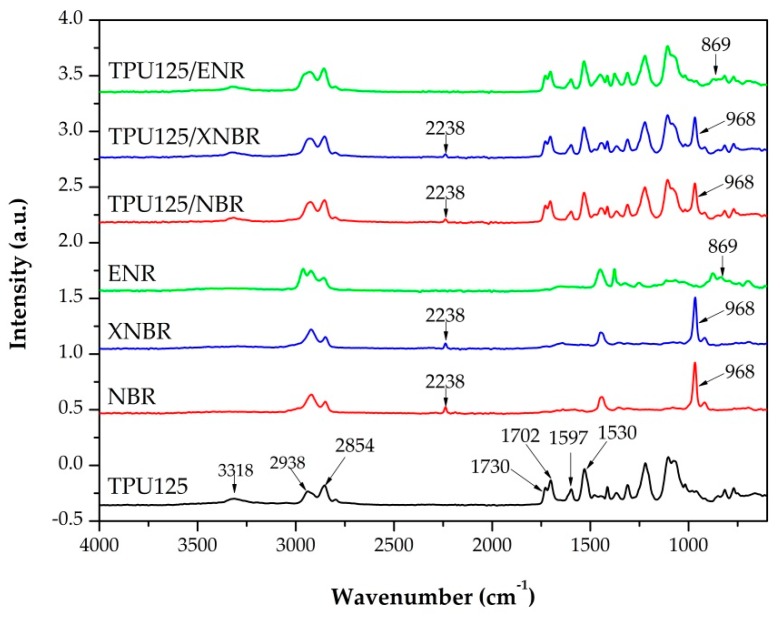
Fourier-transform infrared spectra of the TDVs and TPU125.

We used ATR-FTIR spectroscopy to study the chemical structure of the polymers produced. The recorded spectrum of pure TPU125 and the TDVs containing different rubbers are shown in Figure 11. The spectra contain all of the characteristic peaks (i.e., the >N–H peak at 3318 and 1530 cm^−1^ and the >C=O peaks at 1730 and 1702 cm^−1^) from the urethane linkage. The peak at 1730 cm^−1^ represents the stretching vibration of free >C=O groups, and the peak at 1702 cm^−1^ shows the hydrogen-bonded version. The peak of the isocyanate groups (2275–2250 cm^−1^) was not visible in the spectrum, which indicates that the diisocyanate reacted completely. The peaks at 2938 and 2854 cm^−1^ belong to the >CH_2_ groups of the polymer chains. The aromatic ring in the diisocyanate has an absorption peak at 1597 cm^−1^. On the spectrum of the TPU125/NBR and the TPU125/XNBR, three new peaks appeared, which belong to the rubber phase. The –C≡N peak appeared at 2238 cm^−1^, the CH peak at 1447 cm^−1^, and the =CH_2_ peak at 968 cm^−1^. In the case of TPU125/ENR, one new peak appeared at 869 cm^−1^, which belongs to the epoxy group [24,25,26].

#### 3.2.6. Morphology

##### Atomic Force Microscopy (AFM)

**Figure 12:**
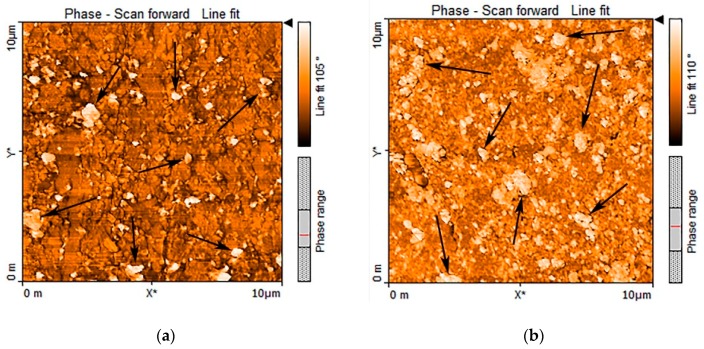

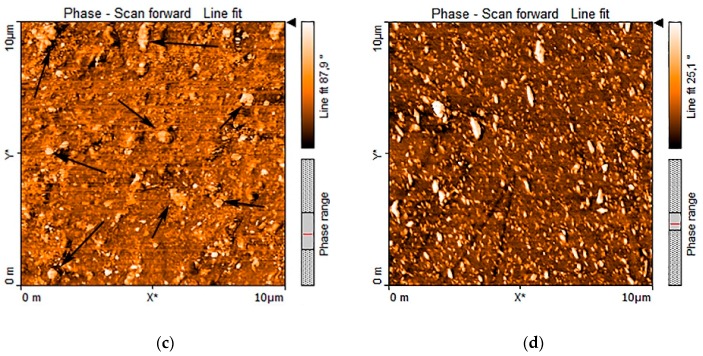
Atomic force microscopy (AFM) phase images (10 μm × 10 μm) of (**a**) TPU125/NBR, (**b**) TPU125/XNBR, (**c**) TPU125/ENR, and (**d**) TPU125.

Figure 12 shows the atomic force microscopy phase images of the TDVs and the neat TPU. The bright parts indicate the hard segments of the TPU due to its high surface energy and modulus, and the dark parts are the soft segment of the matrix [27,28]. Figure 12d shows that phase separation took place in the material. In Figure 12a–c (marked by black arrows), there are 1 to 2 μm morphological units, which are probably the rubber islands distributed in the matrix.

The pictures show that the phase separation occurred in the thermoplastic polyurethane we produced and that we managed to evenly distribute the rubber phase in the matrix.

##### Scanning Electron Microscopy (SEM)

**Figure 13:**
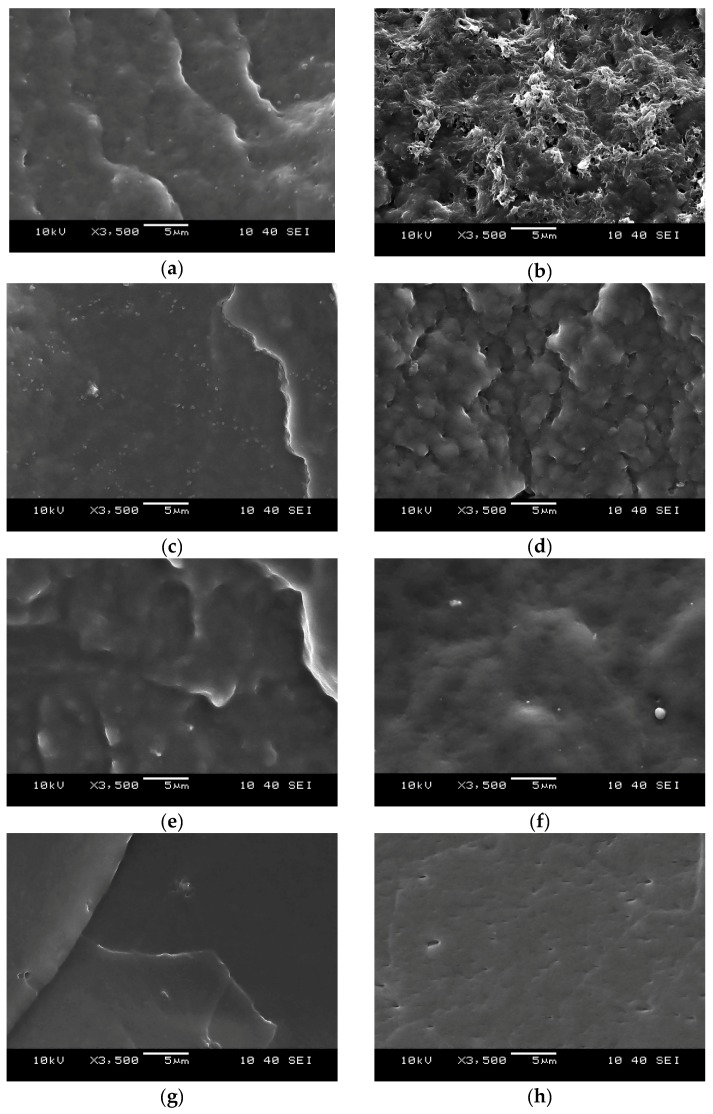
SEM images of the cryo-fractured, and cryo-fractured and etched surfaces of (**a**,**b**) TPU125/NBR, (**c**,**d**) TPU125/XNBR, (**e**,**f**) TPU125/ENR, and (**g**,**h**) neat TPU125.

Figure 13 shows the SEM images of the cryo-fractured (a, c, e, g) and the etched, cryo-fractured (b, d, f, h) surfaces of the samples. Etching with tetrahydrofuran (THF) was successful since there were significant differences between the pairs of pictures. In the case of the neat TPU (Figure 13h), holes with an elongated shape appeared on the surface as a result of etching. This may suggest that the polymerization of the TPU did not finish completely, therefore small regions with lower molecular weights were present in the material. THF dissolved these regions faster than the rest of the TPU, which explains the presence of the holes. Figure 13b shows the TDV containing NBR. The agglomerated particles of the NBR phase can be observed on the surface, and the holes indicate the dissolved TPU matrix. In the case of the TDV containing XNBR (Figure 13d), a stronger interaction may have developed between the XNBR and the TPU phase as a result of a reaction between the carboxyl groups of the XNBR and the isocyanate groups of the MDI component of the TPU, resulting in a chemically bonded thin TPU layer on the surface of the XNBR phase. This assumption is supported by Figure 13d, which shows that a thin layer of TPU remained on the etched surface due to the above-mentioned strong interaction between the XNBR phase and the TPU. Figure 13b,d suggest that the dispersed vulcanized rubber particles are agglomerated, creating a secondary structure. There are weak secondary bonds between these particles, which can reversibly dissociate at elevated temperatures and/or due to high shear. This phenomenon has already been demonstrated for other material pairs (PP/EPDM) [29]. In the case of the TDV containing ENR (Figure 13f), the rubber phase was not visible, which suggests that it was also dissolved. This is most probably because its degree of vulcanization was insufficient to make it insoluble in THF.

## 4. Conclusions

We prepared thermoplastic dynamic vulcanizates with an in situ synthesized TPU matrix and with polar rubbers NBR, XNBR, and ENR. The optimum initial temperature for the internal mixer at which the TPU polymerized and the rubber phase was vulcanized at a sufficient rate without degradation of the materials was 125 °C. The non-isothermal vulcanization and rheological tests confirmed the successful in situ polymerization of the matrix and the dynamic vulcanization of the rubber phase. Based on the AFM phase images, it is likely that the rubber segments were successfully dispersed in the TPU matrix. However, based on the SEM images, these dispersed rubber particles tend to agglomerate and form a secondary quasi-continuous phase structure formed by rubber particles held together only by secondary forces. TDVs have lower hardness and weaker strength characteristics than pure TPU. These TDVs have a higher tensile strength, while their elongation at break is only slightly below that of commercial thermoplastic vulcanizates (TPVs) with similar hardness.

## Figures and Tables

**Table 1:** Mechanical properties of the TPU matrix and the TDVs.

	TPU125	TPU125/NBR	TPU125/XNBR	TPU125/ENR
Elongation at break (%)	377 ± 52	316 ± 35	328 ± 38	447 ± 21
Modulus at 10% (MPa)	1.73 ± 0.05	0.84 ± 0.05	0.77 ± 0.08	0.45 ± 0.08
50% (MPa)	4.35 ± 0.06	1.98 ± 0.06	2.23 ± 0.22	1.04 ± 0.10
100% (MPa)	5.64 ± 0.05	2.92 ± 0.14	3.18 ± 0.23	1.60 ± 0.15
200% (MPa)	8.63 ± 0.09	5.04 ± 0.17	5.79 ± 0.38	2.74 ± 0.26
300% (MPa)	14.24 ± 0.17	7.92 ± 0.16	9.06 ± 0.48	4.09 ± 0.39
Tensile strength (MPa)	24.4 ± 7.8	8.9 ± 0.9	10.8 ± 1.7	6.6 ± 0.9
Hardness (Shore A°)	81.3 ± 0.9	66.4 ± 0.5	64.5 ± 0.7	55.9 ± 0.9

**Table 2:** The glass transition temperatures (*T*_g_) and room temperature storage modulus (*E*′_23 °C_) of the TDVs.

Sample	*T*_g_ (°C)	*E*′_23°C_ (MPa)
TPU125	−15.82	21.82
TPU125/NBR	−11.36	11.51
TPU125/XNBR	−12.05	8.98
TPU125/ENR	−10.23	6.70

**Table 3:** The glass transition (*T*_g_) and the melting (*T*_m_) temperature of the different phases in the TDVs.

Sample	*T*_g, soft_ (°C)	*T*_g, rubber_ (°C)	*T*_g, hard_ (°C)	*T*_m_ (°C)
TPU125	−42.1	—	56.5	211.3
NBR	—	−23.0	—	—
XNBR	—	−22.7	—	—
ENR	—	−24.6	—	—
TPU125/NBR	−43.1	−21.5	53.7	not visible
TPU125/XNBR	−43.0	−20.2	57.6	not visible
TPU125/ENR	−45.5	−22.3	49.9	207.0
